# Cardiovascular changes in the NZB/W F1 mouse model of lupus nephritis

**DOI:** 10.3389/fcvm.2023.1182193

**Published:** 2023-07-24

**Authors:** Romy Böhme, Christoph Daniel, Fulvia Ferrazzi, Miriam Angeloni, Arif Bülent Ekici, Thomas H. Winkler, Karl-Friedrich Hilgers, Ute Wellmann, Reinhard E. Voll, Kerstin Amann

**Affiliations:** ^1^ Department of Nephropathology, Friedrich–Alexander–Universität (FAU) Erlangen–Nürnberg, Erlangen, Germany; ^2^ Institute of Pathology, Friedrich–Alexander–Universität (FAU) Erlangen–Nürnberg, Erlangen, Germany; ^3^ Institute of Human Genetics, University Hospital Erlangen, Friedrich–Alexander–Universität Erlangen–Nürnberg, Erlangen, Germany; ^4^ Divison of Genetics, Department of Biology, Nikolaus–Fiebiger–Center of Molecular Medicine, Friedrich–Alexander–Universität (FAU) Erlangen–Nürnberg, Erlangen, Germany; ^5^ Department of Nephrology and Hypertension, Friedrich–Alexander–Universität (FAU) Erlangen–Nürnberg, Erlangen, Germany; ^6^ Department of Rheumatology and Clinical Immunology, Medical Center–University of Freiburg, Faculty of Medicine, University of Freiburg, Freiburg, Germany

**Keywords:** cardiovascular disease, renal disease, autoimmune disease, systemic lupus erythematosus, interferon-related, IL-15

## Abstract

**Background:**

Patients with systemic lupus erythematosus (SLE), an autoimmune disease, have a higher risk of cardiovascular (CV) disease and death. In addition, up to 40%–50% of SLE patients develop lupus nephritis (LN) and chronic kidney disease, which is an additional CV risk factor. Thus, the individual contributions of LN and other SLE-specific factors to CV events are unclear.

**Methods:**

In this study, we investigated the effect of LN on the development of CV changes using the female NZBxNZW F1 (NZB/W) mouse model of lupus-like disease, with female NZW mice as controls. Standard serologic, morphologic, immunohistologic, and molecular analyses were performed. In a separate group of NZB/W mice, systolic blood pressure (BP) was measured during the course of the disease using tail plethysmography.

**Results:**

Our data show marked CV changes in NZB/W mice, i.e., increased heart weight, hypertrophy of the left ventricle (LV) and septum, and increased wall thickness of the intramyocardial arteries and the aorta, which correlated with the progression of renal damage, but not with the age of the mice. In addition, systolic BP was increased in NZB/W mice only when kidney damage progressed and proteinuria was present. Pathway analysis based on gene expression data revealed a significant upregulation of the response to interferon beta in NZB/W mice with moderate kidney injury compared with NZB mice. Furthermore, IFI202b and IL-6 mRNA expression is correlated with CV changes. Multiple linear regression analysis demonstrated serum urea as a surrogate marker of kidney function and IFI202b expression as an independent predictor for LV wall thickness. In addition, deposition of complement factors CFD and C3c in hearts from NZB/W mice was seen, which correlated with the severity of kidney disease.

**Conclusions:**

Thus, we postulate that the pathogenesis of CV disease in SLE is affected by renal impairment, i.e., LN, but it can also be partly influenced by lupus-specific cardiac expression of pro-inflammatory factors and complement deposition.

## 1. Introduction

Systemic lupus erythematosus (SLE) is an autoimmune disease that results in inflammatory reactions in various tissues, such as the kidney, skin, heart, and nervous system, followed by severe organ damage, i.e., heart failure or end-stage renal disease (ESRD) (1, 2). SLE is characterized by the development of auto-antibodies, such as antibodies to double-stranded (ds) DNA, and activation of the complement system, leading to inflammatory cell infiltration and organ damage (3). The prevalence of SLE varies worldwide; in North America and Europe, the prevalence is approximately 20–50 per 100,000 individuals (4–6), with 9 out of 10 patients being female, mostly of childbearing age (7). Kidney involvement, particularly lupus nephritis (LN), occurs in approximately 40%–50% of these patients and is often the first organ manifestation (8), potentially leading to severe renal impairment and eventually ESRD. In addition, patients with SLE have a significantly increased cardiovascular (CV) risk (9–12) that is increased twofold when SLE patients develop LN (13). CV disease accounts for 20%–31% of deaths and is the most common single cause of death in patients with SLE. The spectrum of CV disease in SLE and the pathomechanisms involved are only partially understood. In particular, the impact of renal disease, i.e., LN, on the development and progression of CV disease remains controversial. This may be important because it has been shown that chronic kidney disease is an important risk factor for CV disease in non-lupus patients (14).

In this study, we investigated the morphological and molecular aspects of CV changes in NZBxNZW F1 (NZB/W) mice, a well-established animal model of lupus-like disease, with particular emphasis on the correlation of CV changes with concomitant LN.

## 2. Materials and methods

### 2.1. Animal model

Female NZW and male NZB mice were purchased from IFFA Credo, France, and maintained and mated in the transgenic mouse facility center (BTE) of the University of Erlangen–Nürnberg (Germany). Female NZBxNZW F1 (NZB/W) mice spontaneously develop a disease closely resembling human SLE (15); in particular, these mice develop a form of proliferative nephritis which is morphologically very similar to human proliferative LN (WHO IV, ISN/RPS IV). In total, 71 female NZB/W F1 mice, along with 16 female NZW mice serving as healthy controls, were maintained in a specific pathogen-free facility in a temperature- and light-controlled environment and had *ad libitum* access to standard chow and water. The regional government (“Regierung von Mittelfranken,” Permit number: 54-2532.1-2-01/07) approved the experimental protocol for the animal studies upon recommendation of the local committee for animal care and use. All animal studies were performed in strict accordance with the German animal welfare law. NZB/W F1 mice were examined weekly for proteinuria as a parameter for renal injury using Albustix® (Bayer AG, Leverkusen, Germany). Since NZB/W F1 mice develop renal disease ranging from mild to severe renal injury, mice were categorized into three different groups based on their severity, namely, mild (*n* = 29), moderate (*n* = 24), and severe (*n* = 18), as described below. To investigate age-dependent cardiac changes, mice were euthanized at various time points, between 17 and 48 weeks of age. At the end of the experiment, mice were anesthetized with isoflurane, blood was collected from the inferior vena cava and perfused with 10% dextran 40 supplemented with 0.02% procaine, and kidneys, hearts, and aortas were collected and fixed in 4% paraformaldehyde buffered in phosphate-buffered saline (PBS) pH 7.4.

### 2.2. Serological evaluation

To analyze serum creatinine, serum urea, and auto-antibodies, blood was transferred to microtainers. After 10 min of incubation at room temperature for clotting, the microtainers were centrifuged at 13,000 rpm for 1.5 min, and the supernatant containing the serum was shock frosted and stored at −20°C. The serum parameters were examined using the Cobas Integra 800 Analyzer (Roche Diagnostics, Mannheim, Germany).

The amount of anti-dsDNA antibodies in the serum of the mice was determined by performing ELISA. For this purpose, 96-well Maxisorp (Nunc, Rochester, NY, USA) plates were coupled in two steps: first with L-lysine, followed by double-stranded calf thymus DNA overnight at 4°C. Non-specific free binding sites were blocked using a solution of 2% fetal calf serum in PBS. Horseradish peroxidase–labeled goat anti-mouse IgG was used as a detection antibody, and the color reaction was induced using 3′,5′5′-tetramethylbenzidine substrate. After stopping the enzymatic reaction with H_2_SO_4_, chromogen was quantified using a microplate reader at *λ* = 495 nm. Serum from mice without lupus was used as negative control.

### 2.3. Morphological evaluation

NZB/W mice of different ages and proteinuria levels, as well as NZW controls, were anesthetized. Blood was extracted, and the animals were perfused with 0.9% NaCl solution. For the histological analysis, hearts, aortas, and kidneys were carefully harvested and weighed. Organs were then fixed in a 4% buffered paraformaldehyde solution for 24 h. After fixation, they were embedded in paraffin and sliced into 1 µm thick cross-sections as described previously (16). For the molecular analysis, a small part of the heart (apex) was sliced, shock frosted, and stored at −20°C. Morphologic analysis was performed on hematoxylin–eosin (HE), Sirius red, periodic acid Schiff (PAS), or immunohistochemical (IHC) stains using light microscopy (various magnifications) and an analysis computer imaging software (Cell^P, Olympus) as described below. For renal damage analysis, kidneys were analyzed for morphologic parameters according to the scoring method of the activity index of Austin in periodic acid Schiff–stained sections (17, 18), which is also used for the analysis of LN in humans. Glomerular changes were evaluated at a magnification of ×400; various scores were obtained in a mean of 50 glomeruli in each animal. The activity index consisted of the following parameters: cell proliferation, hyaline thrombi, cellular crescents, leukocytic infiltration, and fibrinoid necrosis. Each glomerular cross-section was given a score ranging from 0 to 3 for each parameter (0 = no changes, 1 = 0%–25% affected, 2 = 25%–50% affected, and 3 = more than 50% affected). The parameters “cellular crescent” and “fibrinoid necrosis” are multiplied with 2 afterward because these are severe changes. Another parameter is the total mononuclear infiltration in the tubulointerstitium of the whole section, which was also scored 0–3. All scores were then added, resulting in a final score for each animal (minimum of 0 and maximum of 24). To discover in more detail the impact of the degree of renal damage on changes in renal gene expression and CV alterations, LN in NZB/W mice was categorized into mild (score 0–3), moderate (score 4–6), and severe (score >6), resulting in three groups of NZB/W mice.

As parameters of CV disease, the average wall thickness of the left ventricle (LV), septum, aorta, and intramyocardial arteries was quantified by measuring either 10 different positions evenly distributed to the LV, septum, and aorta or the inner and outer diameters of intramyocardial arteries as described previously (19). All morphological analyses were performed in a blinded fashion, i.e., the investigator was unaware of the experimental group the animal belonged to.

### 2.4. Blood pressure and proteinuria measurement

In a separate cohort of eight NZB/W mice (six 13-week-old mice and two 21-week-old mice), systolic blood pressure (BP) was measured weekly using a tail cuff attached to a sphygmomanometer (Perfect Aneroid with automatic valve, ERKA, Bad Tölz, Germany) and the TSE Blood Pressure Monitoring System Software (TSE-Systems, Bad Homburg, Germany). In these mice, proteinuria was also measured weekly in spontaneous urine using Albustix® (Bayer AG). Before the start of the recordings, the mice were trained to this procedure for 4 weeks to prevent false results due to extraordinary stress. To get accustomed to this procedure, we began fixing the mice in a metal restrainer 2–3 times a week following another 3 weeks where we also used the tail cuff and performed test measurements. The weekly results obtained prior to the onset of proteinuria were averaged to a mean BP for each animal; the mean BP at proteinuria was calculated from the mean of the BP at the onset of proteinuria and the BP values of the following week. The final measurement was performed when the mice exhibited advanced LN, and the experiment was terminated at the age of 27–29 weeks.

### 2.5. Immunohistochemistry

Cardiac cross-sections were subjected to IHC staining using antibodies against the T-cell marker CD3 (monoclonal rabbit anti-CD3, clone SP7, Thermo Fisher, Dreieich, Germany), B-cell marker CD20 (monoclonal rabbit anti-CD20, Sigma Aldrich, Deisenhofen, Germany), CD154 (=CD40l) marker for activated T cells (polyclonal rabbit anti-CD154, Santa Cruz Biotechnology, Inc., Santa Cruz, CA, USA), macrophage marker F4-80 (monoclonal rat anti-F4/80, BioRad, Feldkirchen, Germany), vascular cell adhesion molecule-1 (monoclonal rat anti-VCAM-1, BioRad, Feldkirchen, Germany), complement factor D (polyclonal rabbit anti-CFD, Thermo Fisher Scientific, Waltham, MA, USA), complement cleavage product C3d (Abcam, Cambridge, UK), and stable complement cleavage product C3c (polyclonal rabbit anti-C3c, DAKO Deutschland GmbH, Hamburg, Germany). The analysis of positive staining per section was performed next. C3c and C3d were also stained in kidney sections to analyze renal complement deposition. The number of F4/80-positive cells was evaluated using QuPath software and expressed as % positive cells per total cardiac cells. VCAM-1, CFD, and C3d were scored semi-quantitatively: 0 = negative, 1 = up to 25%, 2 = 26%–50%, 3 = 51%–75%, and 4 = more than 75% of area for CFD and C3d or VCAM-1-positive capillaries. In brief, paraffin sections were deparaffined in xylol and rehydrated in graded ethanol (100%–70%). Endogenous peroxidase was inactivated using a solution of 0.3% H_2_O_2_ for 20 min at room temperature. Antigen retrieval was performed using a target retrieval solution (DAKO) and a steam cooker at 120°C for 2.5 min. However, for C3c, antigen retrieval was performed by digestion with protease from *Streptomyces griseus* (Sigma Aldrich, Taufkirchen, Germany), followed by staining using a Ventana Benchmark stainer (Roche AG, Basel, Switzerland). Unspecific binding sites were blocked using an Avidin/Biotin blocking solution (Vector laboratories, Burlingame, CA, USA), the primary antibody was incubated overnight at 4°C or for 1 h at 37°C, and biotinylated secondary antibodies (all from Vector laboratories) were added and incubated for 30 min at room temperature. The ABC solution (Vector laboratories) was added and incubated for 30 min at room temperature. The aminoethyl carbazole solution or DABimmPact were used as chromogenes (Vector laboratories). The analysis of the negative controls was performed by omitting the secondary antibody. Positive cells per high-power field were counted using light microscopy at different magnifications.

### 2.6. Relative gene expression in the heart using microarray analysis and real-time PCR (RT-PCR)

After RNA isolation from frozen hearts using the RNeasy Mini and RNeasy fibrous tissue Mini-Kit (Qiagen, Hilden, Germany) and reverse transcription, cDNA was analyzed in a pilot experiment using an Affymetrix mouse genome 430 2.0 microarray to identify genes and biological pathways involved in the pathogenesis of cardiac changes (NZB/W with moderate renal disease, *n* = 3; NZW controls, *n* = 2). Microarray data analyses were performed within the R/Bioconductor environment v.4.2.2 (20). Raw data were pre-processed using the robust multi-array average (RMA) algorithm through the function *rma* of the Bioconductor affy package v.1.68.0. Differential expression analysis between NZB/W and NZW was performed at the probe set level using the limma package v.3.54.1 (21). Pathway analysis was performed relying on the gene set enrichment analysis (GSEA) approach (22) as implemented in the universal GSEA function *GSEA* of the clusterProfiler package v.4.6.0 (23). As an input list, gene symbols ranked according to decreasing moderated *t*-statistic values were provided. To this aim, probe set IDs were mapped to gene symbols relying on the *mapIds* function of the package AnnotationDbi v.1.60.0, using “CharacterList” as a value for the argument multiVals. Probe set IDs not mapping to any genes or mapping to more than one gene were removed, whereas if different probe set IDs mapped to the same gene, the one with the highest absolute value of the moderated *t*-statistic was chosen to represent the gene. As query gene sets, the Gene Ontology biological process (BP) collection was retrieved from the Molecular Signature Database (MSigDB, https://www.gsea-msigdb.org/gsea/msigdb/mouse/collections.jsp?targetSpeciesDB=Mouse#M5—last accessed on 23 February 2023) by downloading the associated GMT file with gene symbols (v2022.1.Mm). For each pathway, the “leading edge set” is defined as the genes that mostly contributed to enriching the pathway.

In addition, mRNA expression of NZW control animals (*n* = 9) and NZB/W mice with mild (*n* = 16), moderate (*n* = 17), and severe (*n* = 12) renal lesions was measured using real-time PCR (qPCR) (TaqMan Fast 7500, Applied Biosystems), with specific primers for IL-15, IL-6, IL-10, CRP, 18S, IFN-γ, IFI202b, TNF-α, and TGF-β1 (Supplementary Table 1 presents the list of primer sequences). The 12.5 µl reaction mix consists of 6.25 µl 12.5 nM 2× Fast Sybr Green MM buffer (Applied Biosystems), 250 nM forward and reverse primers, 3 µl H_2_O, and 2 µl 12 nM cDNA. Gene expression was calculated using the ^ΔΔ^CT method as described previously (24) in consideration of normalization to housekeeping gene 18S and relative to the gene expression in the heart of the female NZW group.

### 2.7. Statistical analysis

All data were presented as scatter plots showing each single data point and means ± SD using bars and whiskers. Outliers were excluded according to the ROUT outlier test with *Q* = 1%. The numbers of data included per group are given in the figures or table legends. After the values were tested for normal distribution using the Kolmogorov–Smirnov test, data were analyzed using a one-way or two-way analysis of variance (ANOVA) followed by the Bonferroni test. If data were not normally distributed, the Kruskal–Wallis test was used followed by Dunn's multiple comparison test. Spearman’s (skewed distribution) and Pearson’s (normal distribution) correlation analyses were conducted to test the relationship between the parameters. For multiple regression analysis, we used LV wall thickness or relative heart weight as the response variable and age, LN activity index, serum urea, and cardiac expression of IFI202b as predictor variables. All analyses were conducted using IBM SPSS Statistics version 28.0. *p* < 0.05 was considered statistically significant (*p < 0.05; **p < 0.01; ***p < 0.001).

## 3. Results

### 3.1. NZB/W mice developed variable degrees of lupus-like nephritis (LN)

**Figure 1:**
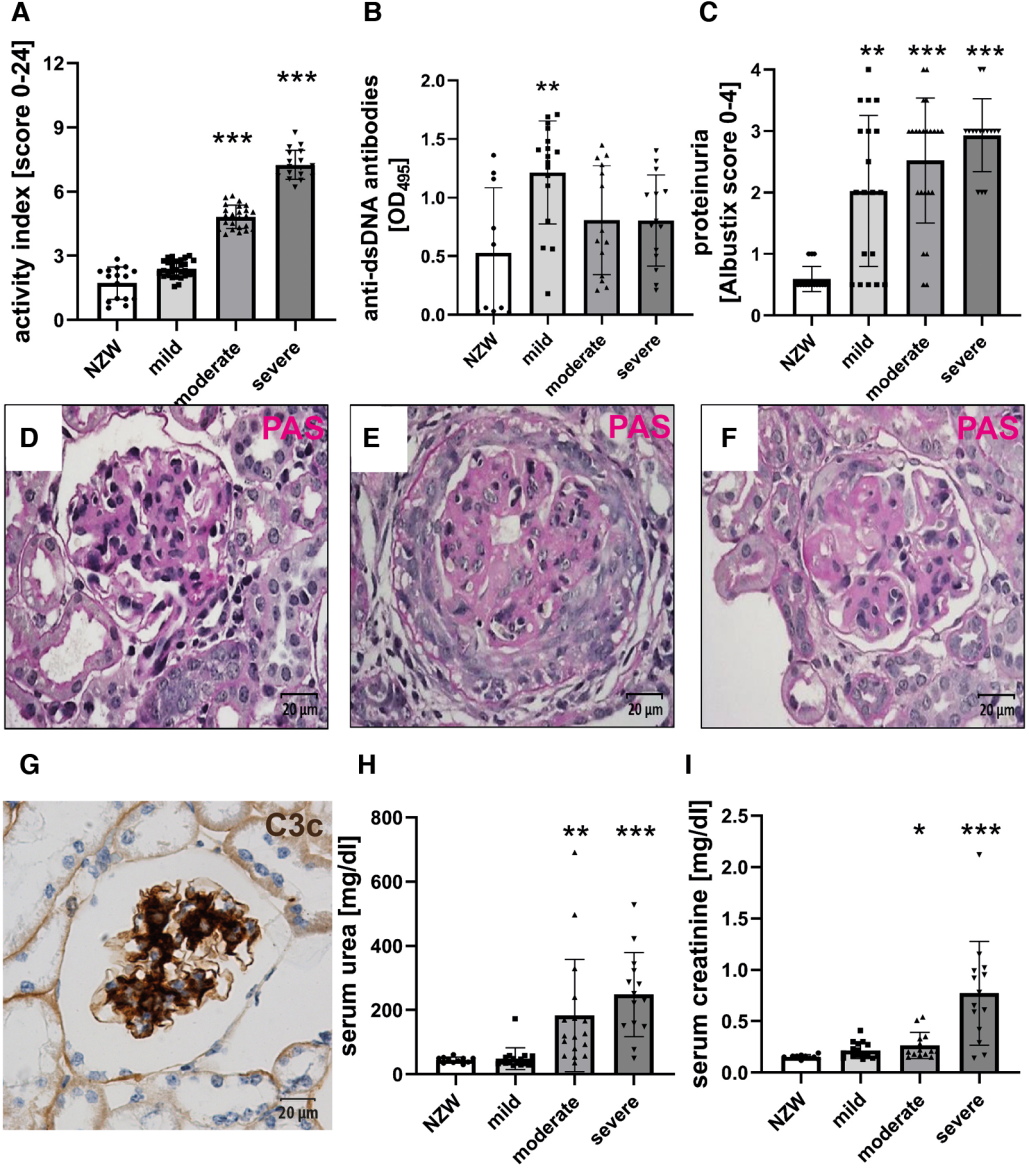
Female NZB/W mice develop lupus-like renal disease. PAS-stained tissue sections were used for the evaluation of renal damage using the Austin activity score (**A**). Sera were analyzed for the presence of anti-dsDNA antibodies using an ELISA (**B**). The urine was analyzed for the occurrence of proteinuria (**C**). Typical renal changes in NZB/W mice include mesangial cell proliferation and matrix accumulation (**D**), cellular crescent formation (**E**), intracapillary hyaline thrombi (**F**), and deposition of complement C3c (**G**), as shown by immunohistochemistry. Sera were analyzed for urea (**H**) and creatinine (**I**). Asterisks marked significant differences compared with the NZW control group. *p < 0.05, **p < 0.01, and ***p < 0.001.

NZB/W F1 females were analyzed at various ages to investigate age-related changes, ranging from 17 to 48 weeks, with a mean age between 28 and 30 weeks (Table 1). Using the Austin activity score of LN, female NZB/W mice were divided into three groups based on their severity, namely, mild, moderate, or severe (Figure 1A), to investigate whether CV changes in LN were dependent on the severity of renal damage. Body weight and relative kidney weight in NZB/W mice were comparable to those of the NZW control group but showed minor differences between the NZB/W groups (Table 1). In NZB/W mice, LN was characterized by the production of anti-dsDNA antibodies (Figure 1B) and the development of proteinuria. The proteinuria levels slightly increased with the severity of the disease but showed no significant differences within the nephritic groups (Figure 1C). Similar to human LN, NZB/W mice exhibited typical glomerular changes such as mesangial cell proliferation and matrix expansion (Figure 1D), cellular crescent formation (Figure 1E), intracapillary hyaline thrombi (Figure 1F), and C3c and C3d complement deposition (Figure 1G and Supplementary Figure S1). In addition, variable amounts of inflammatory cells and complement C3d deposition (Supplementary Figure S1) were observed in the tubulointerstitium and perivascular space. Serum urea and creatinine, which are surrogate markers of renal function, were comparable between the mild LN group and the NZW control group and were significantly elevated in the moderate LN group and especially in the severe LN group (Figures 1H,I). Interestingly, the degree of LN, as assessed by the Austin score (*r* = 0.111, *p* = 0.31), and serum creatinine, as a marker of renal function (*r* = 0.038, *p* = 0.779), were not dependent on the age of the NZB/W mice studied.

**Table 1:** Animal characteristics of studied NZW and NZB/W mice. Age, body weight, and relative kidney weight of NZW controls and NZB/W mice were shown in groups with different extent of renal damage at the end of the experiment.

	Age (weeks)	Body weight (g)	Kidney-to-body weight (mg/g)
NZW controls	30.6 ± 12.4 (*n* = 15)	30.5 ± 3.1 (*n* = 15)	7.5 ± 0.9 (*n* = 15)
NZB/W mild renal damage	27.9 ± 4.4 (*n* = 29)	33.4 ± 3,5^c^ (*n* = 29)	6.6 ± 0.8 (*n* = 29)
NZB/W moderate renal damage	28.1 ± 4.1 (*n* = 24)	31.6 ± 3.8^c^ (*n* = 23)	7.9 ± 2.1 (*n* = 23)
NZB/W severe renal damage	28.4 ± 3.8 (*n* = 18)	27.4 ± 5.7^a,b^ (*n* = 18)	8.5 ± 1.1 (*n* = 18)

^a^ *p* < 0.05 vs. NZB/W mild renal damage.

^b^ *p* < 0.05 vs. NZB/W moderate damage.

^c^ *p* < 0.05 vs. NZB/W severe damage.

### 3.2. CV changes in experimental lupus-like disease are related to the severity of renal disease

**Figure 2:**
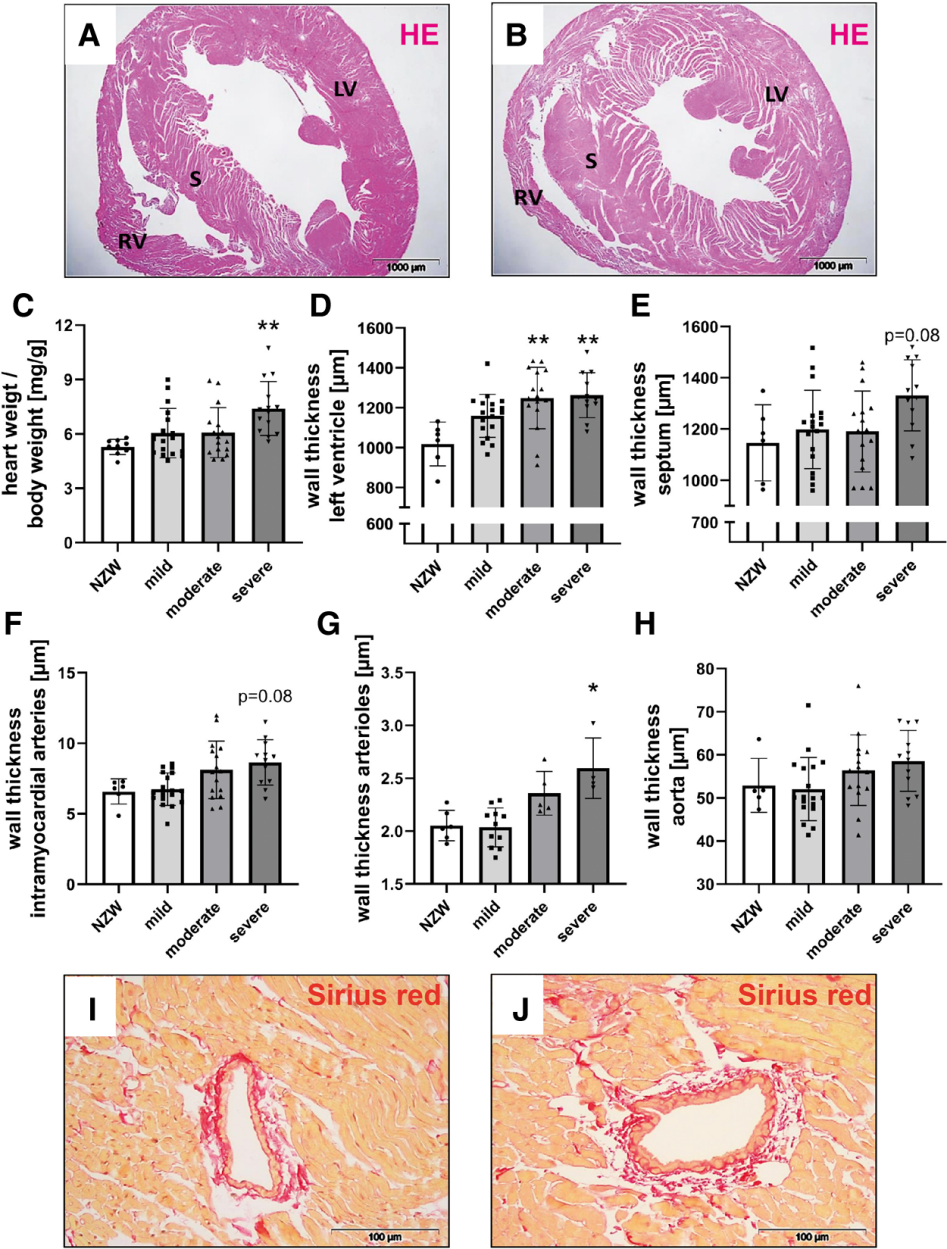
Cardiovascular changes in NZB/W mice with lupus-like disease. Hematoxylin–eosin (HE)–stained cardiac sections of NZW (**A**) and NZB/W (**B**) mice were analyzed for characteristic changes. The following parameters were analyzed: relative heart weight (**C**), left ventricular wall thickness (**D**), septum wall thickness (**E**), intramyocardial wall thickness (**F**), and aorta wall thickness (**G**). Representative pictures of Sirius red–stained intramyocardial arteries in NZW (**I**) and NZB/W (**J**) mice were shown. Asterisks marked significant differences compared with the NZW control group. **p < 0.01.

CV changes were analyzed using cardiac histological sections from NZB/W mice with lupus-like disease (Figure 2B) and healthy NZW controls (Figure 2A). While a significant increase in relative heart weight could only be observed in animals with severe kidney damage (Figure 2C), LV wall thickness was already significantly increased in mice with moderate kidney damage (Figure 2D). Even in animals with only mild kidney damage, a tendency toward a thickened LV wall was observed compared with the NZW control group (Figure 2D). Furthermore, intramyocardial arteries (Figures 2F,I,J), arterioles (Figure 2G), and the aortic vessels (Figure 2H) also showed a tendency toward increased wall thickening depending on the severity of LN.

**Figure 3:**
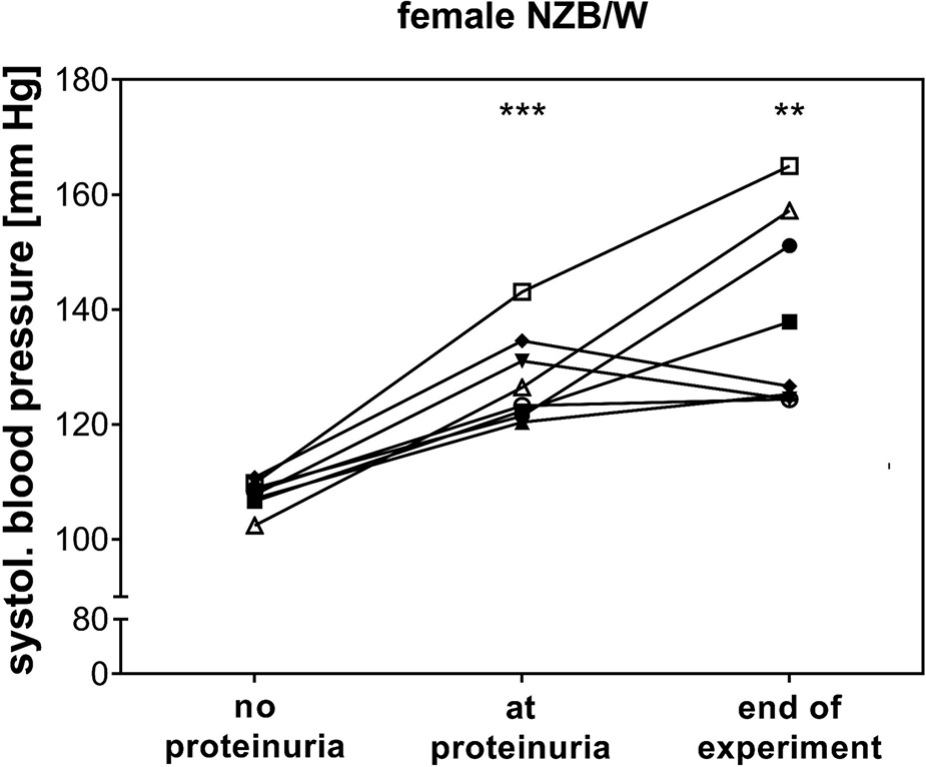
In female NZB/W mice with lupus-like disease, blood pressure increased at the onset of proteinuria. Mean systolic blood pressure (BP) before the onset of proteinuria (no proteinuria), at the onset of proteinuria, and at the end of the experiment was shown for each individual animal (*n* = 8). BP was significantly increased at the onset of proteinuria (***p < 0.001) and at the end of the experiment (**p = 0.01) compared with mean BP in the NZB/W mice before the onset of proteinuria.

Correlation analyses showed that relative heart weight correlates strongly with serum creatinine (*r* = 0.672, *p* < 0.001) and proteinuria (*r* = 0.592, *p* < 0.001) and also with serum urea (*r* = 0.452, *p* < 0.01) and LN activity score (*r* = 0.346, *p* < 0.01) (Supplementary Table S4). The study also found lower, but significant, correlations between LV wall thickness and thickness of intramyocardial arterial walls with LN activity score, serum urea, and serum creatinine (Supplementary Table S4). In contrast, aortic wall thickness significantly correlated only with LN activity score, and septum wall thickness only with proteinuria (Supplementary Table S4). To explore the effect of selected candidate renal predictors on CV changes, we performed multiple linear regression analyses. More specifically, relative heart weight and LV wall thickness were used, in turn, as response variables, and LN activity score, serum urea, age, and IFI202b expression were used as predictors. Results showed that serum urea was an independent predictor (*p* = 0.015) for relative heart weight (Table 2), whereas both serum urea (*p* = 0.036) and IFI202b expression (*p* = 0.01) were independent predictors for LV wall thickness (Table 3). The observed CV changes were independent of the age of the animals (Tables 2, 3) but are associated with the autoimmune disease itself. Using weekly tail cuff BP measurements, we demonstrated that systolic BP was increased in NZB/W mice with the progression of renal disease. At baseline, systolic BP in the eight NZB/W mice had a median of 108 mmHg (min. 102, max. 111). In the presence of proteinuria (≥300 mg/dl = Albustix +++), BP increased to a median of 125 mmHg (min. 120, max. 143) and further increased toward the end of the experiment to a median of 132 mm Hg (min. 124, max. 165) (Figure 3).

**Table 2:** Results from linear regression analysis using relative heart weight as a response variable and LN activity score, serum urea, age, and IFI202b expression as predictors (*n* = 39).

	Regression coefficient	Std. error	*T*	*p*-value
Intercept	8.076	3.602	2.242	**0**.032 (*)
LN activity score (0–24)	−0.222	0.256	−0.869	0.391
Serum urea (mg/dl)	0.011	0.004	2.572	**0**.015 (*)
Age (weeks)	−0.016	0.064	−0.246	0.807
Card. IFI202b expression (^ΔΔ^CT-value)	−0.149	0.212	−0.700	0.489

LV, left ventricular; LN, lupus nephritis; Std. error, standard error; *T*, *t*-value.

Significant *p*-values are highlighted in bold.

* *p* < 0.05.

**Table 3:** Results from linear regression analysis using LV wall thickness as a response variable and LN activity score, serum urea, age, and IFI202b expression as predictors (*n* = 34).

	Regression coefficient	Std. error	*T*	*p*-value
Intercept	440.729	251.366	1.753	0.090
LN activity score (0–24)	−5.517	16.910	−0.326	0.747
Serum urea (mg/dl)	0.625	0.285	2.194	**0**.036 (*)
Age (weeks)	5.721	4.073	1.405	0.171
Card. IFI202b expression (^ΔΔ^CT-value)	61.164	16.213	3.773	**0**.001 (**)

LV, left ventricular; LN, lupus nephritis; Std. error, standard error; *T*, *t*-value.

Significant *p*-values are highlighted in bold.

* *p* < 0.05, **p < 0.01.

### 3.3. CV changes in NZB/W mice were accompanied by a specific intracardiac interferon signature

**Figure 4:**
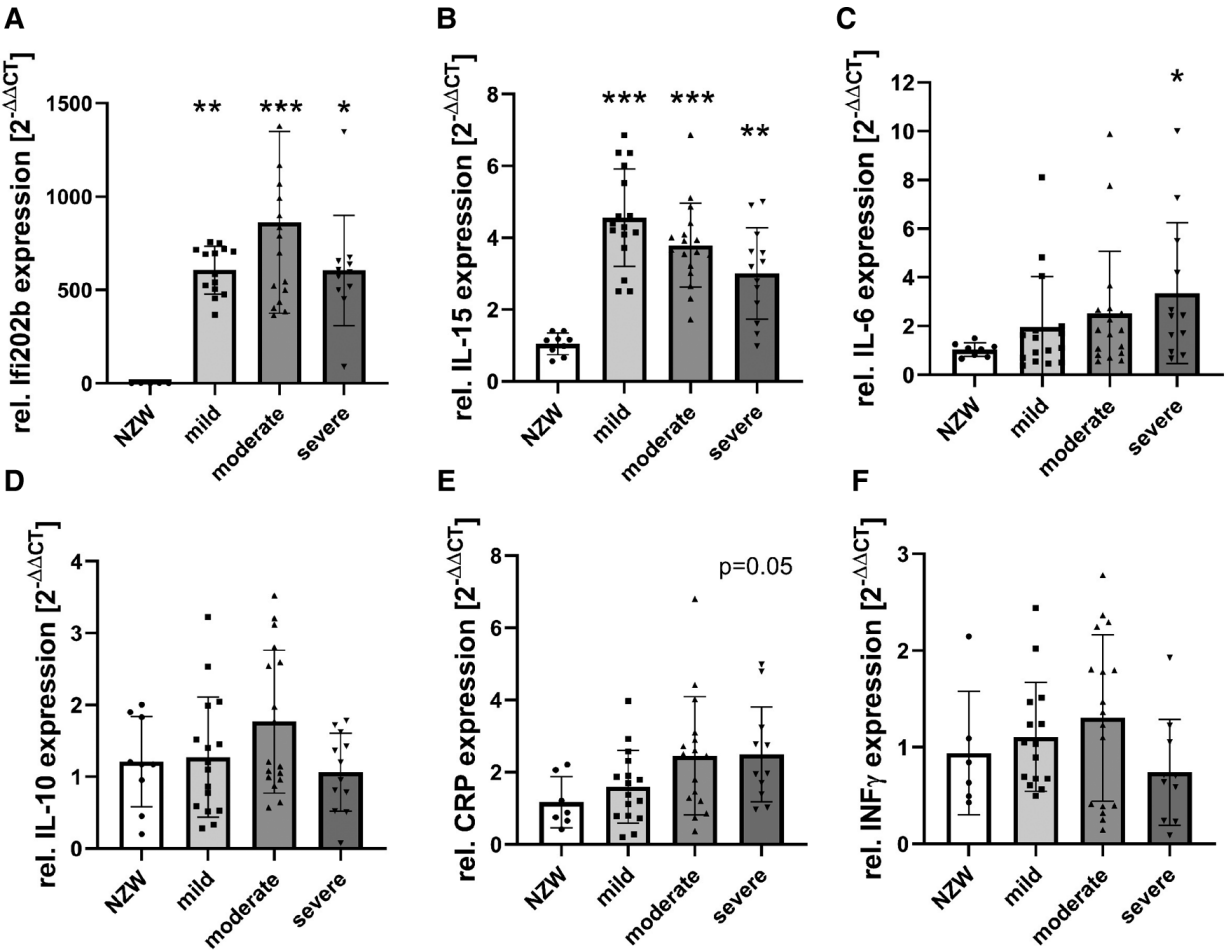
mRNA expression of pro-inflammatory cytokines and factors in NZB/W mice with mild, moderate, and severe renal damage compared with NZW controls. After isolation of mRNA from cardiac tissue of NZB/W and NZW mice, quantitative real-time PCR was performed for IFI202b (**A**), IL-15 (**B**), IL-6 (**C**), IL-10 (**D**), C-reactive protein (CRP, **E**), and IFN-γ (**F**). Data were analyzed using the DDCT method and the NZW group as controls. Data are expressed as fold change NZB/W to NZW controls. Asterisks marked significant differences compared with the NZW control group. *p < 0.05, **p < 0.01, and ***p < 0.001.

**Figure 5:**
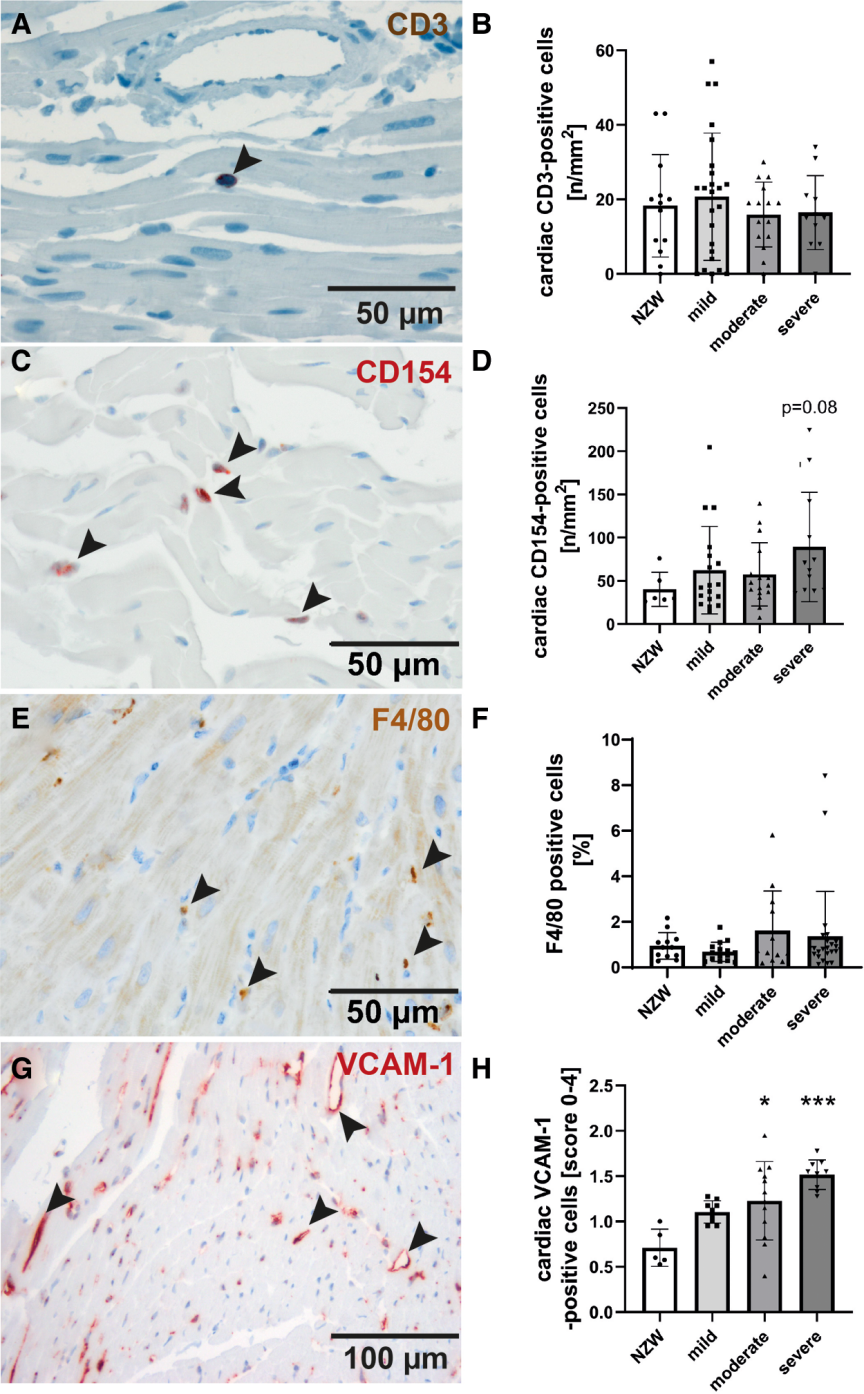
Evaluation of markers of cardiac inflammation and endothelial activation. Immunohistochemistry was used to detect CD3-positive T-cells (**A, B**), CD154-positive activated T-cells (**C, D**), F4/80-positive macrophages (**E, F**) and the vascular cell activation marker-1 (VCAM-1) (**G, H**) and analyzed by counting of positive cells (**B, D, F**) or using a semi-quantitative score (H). Significant differences compared to NZW control group were marked by asterisks. * p<0.05, ***p<0.001.

**Figure 6:**
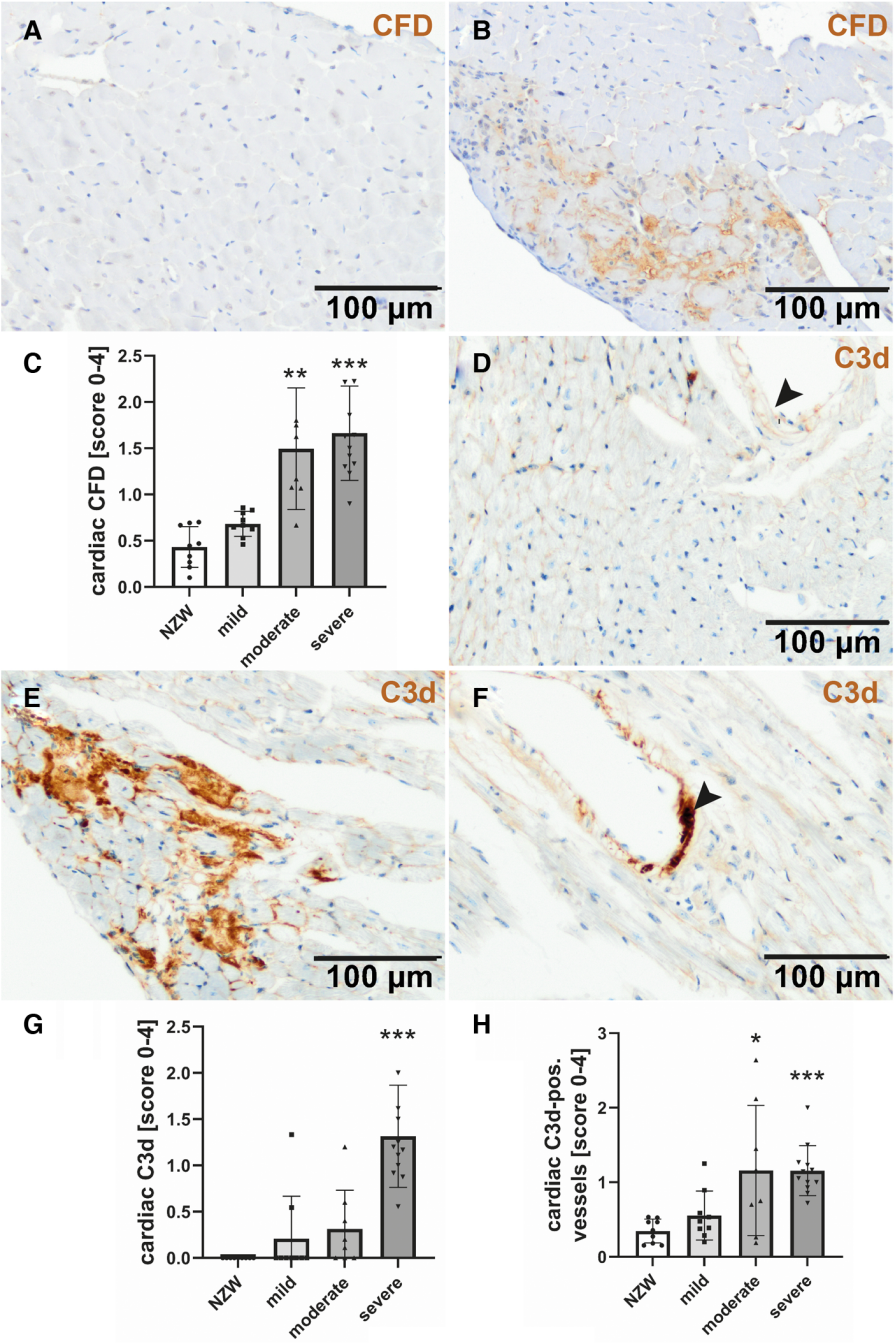
Cardiac complement deposition in NZB/W mice with mild, moderate, and severe renal damage compared with NZW controls. Immunohistochemistry was used to detect complement factor D (CFD) in NZW controls (**A**) and NZB/W mice with severe renal injury (**B**), followed by semi-quantitative scoring in all groups (**C**). C3c was rarely found in NZW (**D**) but was markedly deposited in and around cardiomyocytes (**E**) and cardiac vessels (**F**) of mice with severe renal injury. Semi-quantitative evaluation of cardiac (**G**) and vascular C3c deposition (**H**) was shown. Asterisks marked significant differences compared with NZW control group. *p < 0.05, **p < 0.01, and ***p < 0.001.

To identify possible mediators of CV changes in experimental lupus-like disease, we investigated cardiac mRNA expression profiles in a pilot microarray experiment that compared three mice with moderate renal injury (NZB/W) with two age-matched NZW controls. GSEA identified 70 significantly dysregulated pathways (*p*-adjusted < 0.01), of which 59 were upregulated and 11 downregulated (Supplementary Table S2). The top upregulated pathway was “GOBP_RESPONSE_TO_INTERFERON_BETA,” with interferon type 1 inducible gene (IFI202b) being the most upregulated leading edge gene of the pathway (Supplementary Table S3). IFI202b is known to be expressed in B cells and is thought to be involved in the development of SLE. Quantitative gene expression analyses of pro-inflammatory cytokines and factors in the heart of NZB/W mice and NZW control mice confirmed a significant and strong upregulation (>500-fold) of IFI202b (Figure 4A). The upregulation of IFI202b expression was independent of the severity of LN (Figure 4A) but correlated with different CV markers, including increased wall thickness of the LV, septum, and aorta (Supplementary Table S5). Interestingly, cardiac IFI202b and IL-6 expression correlated well with the renal expression of these genes (*r* = 0.942 and *r* = 0.592 with *p* < 0.01) (Supplementary Table S6). However, using immunohistochemistry, we failed to detect CD20-positive B cells in cardiac tissue sections, while B cells were prominent in high numbers in foci of interstitial inflammation in kidney sections of NZB/W mice (data not shown). Other top upregulated pathways include immune response–related pathways (i.e., “GOBP POSITIVE REGULATION OF IMMUNE RESPONSE” and “GOBP ADAPTIVE IMMUNE RESPONSE”), containing several interleukins in the leading edge sets. In particular, IL-15 was the most upregulated interleukin assayed with the microarrays. Based on quantitative gene expression analyses, it was significantly upregulated, up to fourfold, in cardiac tissue of NZB/W mice, showing a tendency to a lower expression with more severe kidney damage in mice (Figure 4B), but was not correlated with any observed CV alteration. In contrast, cardiac mRNA expression of IL-6 was only slightly increased in NZB/W mice with severe kidney damage (Figure 4C). However, the level of cardiac IL-6 expression was positively correlated with the wall thickness of the LV, septum, and intramyocardial arteries (Supplementary Table S5). Other cytokines and pro-inflammatory factors, such as IL-10 (Figure 4D), C-reactive protein (CRP, Figure 4E), IFN-γ (Figure 4F), and TGF-β and TNF-α (data not shown), were not significantly upregulated in NZB/W mice compared with NZW controls and were weakly correlated, with changes of single CV parameters (Supplementary Table S5). Compared with the correlation between renal damage and function with CV damage, the cardiac expression of pro-inflammatory factors shows a less frequent and weaker correlation with CV changes (Supplementary Tables 4, 5). Using immunohistochemistry, we only detected low numbers of CD3-positive T cells in cardiac tissue sections, without significant differences in numbers between the investigated groups (Figures 5A,B). CD154 is predominantly expressed on activated T cells and tended to have higher numbers only in the hearts of NZB/W mice with severe renal damage (Figure 5D). Interestingly, endothelial VCAM-1 was significantly upregulated in cardiac tissue sections of NZB/W mice with moderate and severe renal damage (Figures 5E,F), indicating increased cardiac endothelial activation in NZB/W mice with lupus-like disease. Since complement factors are important components of the upregulated pathway “GOBP POSITIVE REGULATION OF IMMUNE RESPONSES” in NZB/W mice in our pilot microarray, we also examined complement deposition in the heart. In hearts from NZW control mice, neither complement factor B-interacting factor D (CFD) (Figures 6A,C) nor the stable fragment of central complement factor C3d (Figures 6D,G) could be detected by immunohistochemistry. In contrast, in hearts from NZB/W mice with moderate and severe renal injury, CFD (Figures 6B,C) and C3d (Figures 6E,G) were significantly deposited in cardiomyocytes and the interstitial space. In addition, C3d was also increasingly deposited in cardiac vessels (Figures 6F, arrowhead, 6H) in these animals, but not in vessels of NZB/W mice (Figure 6D, arrowhead).

## 4. Discussion

### 4.1. CV changes in a mouse model of LN

SLE is an autoimmune disease that affects multiple organs, including the skin, kidneys and CV system, and particularly the heart. CV disease is a major clinical problem in patients with SLE (9–12), with cardiac hypertrophy and advanced coronary artery disease being part of the clinical spectrum of this disease. The pathogenesis and exact morphology of CV changes in SLE, as well as the potential correlation to concomitant renal disease and BP changes, have not been investigated in detail so far. Therefore, we aimed to describe the spectrum and course of CV disease in different degrees of renal disease in female NZB/W mice, a well-established experimental model of lupus-like disease.

We found that CV alterations, such as increased heart weight, LV hypertrophy, thickened interventricular septum, and increased wall thicknesses of the intramyocardial arteries and the aorta, were highly correlated with the severity and progression of LN, but not with the age of the animals. These findings suggest that CV changes in SLE may not be entirely specific to lupus alone, but rather can be influenced by renal disease, which is consistent with findings in patients with other renal diseases (25) as well as in rats with experimental renal insufficiency (26). In our multiple linear regression analyses, we demonstrated that in the NZB/W LN model, serum urea, as a surrogate marker of renal function, was an independent predictor of two cardiac parameters, namely, LV wall thickness and relative heart weight. In contrast, IFI202b expression was only an independent predictor of LV wall thickness. Thus, renal function is an important, but not the only, factor influencing cardiac changes in LN. A meta-analysis of SLE patients found that the risk of developing CV complications was doubled in SLE patients with LN compared with those without LN (13). Previous studies in uremic non-lupus patients and rats with induced renal damage also showed an increase in the wall thickness of intramyocardial arteries and the aorta (27–29). In addition, we found a specific interferon signature, which could be involved in the pathogenesis of CV disease in this animal model. Several genes known to play a role in the development of cardiac hypertrophy, such as syndecan-4 (30), or in cardiac injury, such as troponin T2 (31), were also upregulated in the hearts of NZB/W female mice. These genes may be involved in mediating CV damage, but the factors by which they were upregulated remain open and need to be investigated in future studies. CV changes occur in various models of SLE but differ in expression and type (32, 33). Our own preliminary unpublished studies in the yaaFCγR2b lupus model, which develops less renal damage than NZB/W mice, also showed CV changes with a tendency to be more severe in animals with marked renal damage, suggesting that CV changes are not limited to the NZB/W model and may be dependent on renal damage.

An additional effect of BP on CV disease is certainly possible but cannot be formally addressed in the present study due to the study design. However, we observed that systolic BP was also independent of age but increased when the mice developed proteinuria. It further increased with the progression of renal failure. This finding is in line with earlier findings in mice with partial nephrectomy, which showed a significant increase in the systolic BP (to approx. 160 mmHg) in comparison to controls (34). In the studies by Rudofsky et al. (35) and Ryan (36), systolic hypertension (approx. 140 mmHg) in NZB/W F1 mice could only be observed in the presence of renal damage, while mice without glomerular lesions had a normal BP (36). However, in these studies, other mouse models (BXSB and MRL/llpr mice) did not develop hypertension despite progressive renal damage. Furthermore, SLE patients also developed hypertension in the absence of a diagnosed LN (37, 38). Therefore, the impact of renal damage on the development of hypertension in SLE remains controversial.

### 4.2. Expression of relevant inflammatory cytokines and complement deposition in the heart of NZB/W is selective

A relevant pathogenetic role for CV disease in SLE has been suggested for systemic or local inflammation (39), which could be induced by various cytokines. Therefore, gene expression analyses for TNF-α, TGF-β, IFN-γ, CRP, IFI202b, IL-6, IL-10, and IL-15 were performed by RT-PCR. Inflammation could be detected by the production of “acute phase proteins” such as C-reactive protein (CRP). CRP is upregulated by pro-inflammatory cytokines, particularly interleukin-6 (IL-6) and TNF-α (40–42). TNF-α is associated with inflammatory and destructive processes and has been shown to be one of the most important pro-inflammatory cytokines in rheumatic diseases and—possibly—in SLE (43). In the study by Pomara et al. (44), TNF-α expression was detected in the cardiac tissue of an SLE patient. Other cytokines that play an important role in SLE (45) are interferon-γ (IFN-γ), IL-6, and IL-10 (46, 47). In the study by Tomita et al. (48), the cardiac gene expression of IL-10, IFN-γ, IL-6, TNF-α, and TGF-β was investigated in 6- and 26-week-old MRL/lpr mice (another experimental model of lupus-like disease). In this study (48), the gene expression of IL-10 and IFN-γ was increased in both 6- and 26-week-old mice compared with CBA/J control mice, and IL-6 expression was upregulated in 26-week-old MRL/lpr mice. In our study, cardiac CRP and IL-6 gene expression tended to be upregulated in NZB/W with progressive renal damage. In addition, it was correlated with CV disease. In contrast, the expression of IL-10, IFN-γ, and TGF-β and TNF-α was comparable in the NZB/W groups and NZW controls, showing little or no correlation with CV disease. The above study (48) also confirms our IL-6 expression data, although the degree of renal damage was not analyzed. In our study, no significant difference in TNF-α and TGF-β gene expression was observed in experimental lupus-like disease compared with control mice. In contrast, the gene expression of IL-10 and IFN-γ differed in our study and in the study of Tomita et al. (48), suggesting that the model of lupus-like disease may also be relevant. Of note, we did not find a difference in cardiac TNF-α gene expression, which is known to induce IL-6 production in rheumatic arthritis and inflammatory diseases (49). TNF-α has also been discussed as a marker for myocardial disease (50) and has been shown to be produced in cardiac myocytes and endothelium. However, it remains unclear which cell type is responsible for this expression (51, 52). Another study suggested that TNF-α may play a role in myocardial dysfunction in a mouse myocarditis model (52). In our study, cardiac TNF-α gene expression was detectable but only slightly differed in mice with and without morphological CV changes, so the role of TNF-α remains controversial.

On immunohistochemistry, we found no B cells and only a few T cells and macrophages in the heart without differences between groups. However, the changes in cardiac cytokine gene expression described above may be the result of a few infiltrating mononuclear cells that do not show up on histology (53). Alternatively, cardiac myocytes themselves are known to express relevant inflammatory cytokines. For example, a recent study showed that IL-6-cadherin 11 signaling between fibroblasts and cardiomyocytes promoted ventricular remodeling, which was confirmed in both animal and *in vitro* experiments (54). In addition, we observed intracardiac mRNA expression of CFB associated with significant deposition of CFD and C3d in the hearts of NZB/W mice with moderate to severe renal injury. Complement deposition has been described in human studies of dilated cardiomyopathy (55) and SLE (44). Pronounced complement deposition in the kidneys, depending on the degree of renal injury, was typical for LN and also detected in our NZB/W model, indicating systemic complement activation. Whether complement factors reflect or cause cardiac damage is unclear. Using RT-PCR, we found that IFI202b, which has been discussed as a lupus susceptibility gene locus (47), was very highly expressed in NZB/W mice compared with NZW controls, with no difference between NZB/W groups with different levels of LN. However, IFI202b expression is correlated with LV wall thickness, but not with relative heart weight, suggesting a potential role of lupus-induced cardiac inflammation in the pathogenesis of CV disease. In addition, the expression of IL-15 showed a similar pattern (although the expression level was not as high as that of IFI202b). Interestingly, the gene expression of IL-15 decreased with the progression of LN. IL-15 is expressed in various tissues and has known effects on the proliferation of B cells, activated T cells, and natural killer cells (56–58). It is also a myokine involved in regulatory processes in heart failure (59). In a patient with SLE, Pomara et al. (44) described the IL-15 gene expression in cardiac myocytes; similarly, elevated serum levels of IL-15 have been reported in SLE patients (60, 61). Therefore, although IL-15 is not correlated with CV disease, it is conceivable that IL-15 may play a pathogenetic role in CV disease in SLE, but this could not be further investigated in our study.

## 5. Conclusions

We have shown that the progression of lupus-like nephritis in female NZB/W F1 mice is paralleled by an increase in systolic BP and correlates with some CV changes in experimental lupus-like disease. In particular, the cardiac expression of IFI202b and IL6 is also correlated with CV changes, although to a lesser extent, suggesting that these changes may be caused by renal damage and are also influenced by lupus-specific cardiac expression of pro-inflammatory factors.

## Data Availability

The datasets presented in this study can be found in online repositories. The names of the repository/repositories and accession number(s) can be found in the article/Supplementary Material.
