# Supplementary material for: Cardiovascular changes in the NZB/W F1 mouse model of lupus nephritis
Source: Front Cardiovasc Med. 2023 Jul 24;10:1182193. doi: 10.3389/fcvm.2023.1182193 (PMC10405627; doi:10.3389/fcvm.2023.1182193)
Supplement: Supplementary file 1 [file Table1.docx]

Supplementary Material

**Cardiovascular changes in the NZB/W F1 mouse model of lupus nephritis**

**Romy Böhme^1+^ ; Christoph Daniel^1+^, Fulvia Ferrazzi^1,2^, Miriam Angeloni^2^, Arif Bülent Ekici^3^, Thomas H. Winkler^4^, Karl-Friedrich Hilgers^5^, Ute Wellmann^4^, Reinhard E. Voll^6^, Kerstin Amann^1*^**

*** Correspondence:** Prof. Dr. Kerstin Amann e-mail: [Kerstin.Amann@uk-erlangen.de](mailto:Kerstin.Amann@uk-erlangen.de)

# Supplemental Table 1: Primer sequences used in real-time PCR.

# 18S was used as *housekeeping gene*.

| **Gene name** | **5‘-3‘ Sequence forward** | **5‘-3‘ Sequence reverse** |
| --- | --- | --- |
| **INF-γ** | ATTGCCAAGTTTGAGGTCAACA | TGGTGGACCACTCGGATGA |
| **Ifi202b** | CGGCTTGAAGAACTCAATCAAAA | CCTTAAGTTACGGTTGGACATTGC |
| **IL-15** | AACCCATGTCAGCAGATAACCA | GCCATCCATCCAGAACTCTTCT |
| **IL-10** | CAAAGGACCAGCTGGACAACA | GCAACCCAAGTAACCCTTAAAGTC |
| **IL-6** | TCGGAGGCTTAATTACACATGTTC | TGCCATTGCACAACTCTTTTCT |
| **TNF-α** | GACCCTCACACTCAGATCATCTTCT | CCACTTGGTGGTTTGCTACGA |
| **TGF-β** | TGACGTCACTGGAGTTGTACGG | GGTTCATGTCATGGATGGTGC |
| **CRP** | CTCTGGTGCCTTCTGATCATGA | GGAAGTATCTGACTCCTTGGGAAA |
| **18S** | ttgattaagtccctgccctttgt | cgatccgagggcctcacta |

**For Data from Suppl. Table 2 and 3 see separate Excel files.**

**Suppl. Table 2: Significantly enriched (adjusted p-value < 0.01) pathways based on gene expression analysis comparing NZB/W with moderate lupus disease (n=3) with age-matched NZW (n=2) in a pilot microarray analysis.** For each pathway the table shows: the associated Gene Ontology identifier (GO_ID), the number of genes included in the pathway (setSize), its degree of enrichment (enrichmentScore (ES)), the normalized enrichment score (NES), the p-value (pvalue), the p-value adjusted with Benjamini-Hochberg correction (p.adjust), and the set of genes that mostly contributed to make the pathway enriched (leading_edge_genes). Pathways are sorted in ascending order according to the column p.adjust. ES (or NES) > 0 indicates a pathway up-regulated (i.e. positively enriched) in the NZB/W group whereas an ES (or NES) < 0 indicates a pathway down-regulated in the NZB/W group (i.e. enriched in the NZB group). The raw data from the pilot microarray study are available with the accession number GSE226308 from the NCBI Gene Expression Omnibus (GEO) database (https://www.ncbi.nlm.nih.gov/geo/).

**Suppl. Table 3: Results of differential expression analysis comparing NZB/W with moderate lupus disease (n=3) with age-matched NZW (n=2) in a pilot microarray analysis.** For each probe set ID the table shows: the corresponding gene symbol(s), its logFC (logarithm to the base 2 of the fold change), its average log2-expression (AvgExpr), the moderated t-statistic (t), the nominal p-value of the moderated t-statistic (P.Val) and the p-value adjusted with Benjamini-Hochberg correction (adj.P.Val). Results are sorted in ascending order according to the column adj.P.Val. In the column Gene Symbol, the value “NA” is given for probe set IDs not mapping to any gene symbols, whereas for probe set IDs mapping to multiple gene symbols all the corresponding genes are reported and separated through the character “//” . The raw data from the pilot microarray study are available with the accession number GSE226308 from the NCBI Gene Expression Omnibus (GEO) database (https://www.ncbi.nlm.nih.gov/geo/).

Supplemental Table 4. Pairwise correlation analyses of cardiovascular changes with renal damage and kidney function and age in female NZB/W mice with lupus disease.

|  | **lupus nephritis activity score**  **[0-24]** | **urea (serum) [mg/dl]** | **creatinine (serum) [mg/dl]** | **proteinuria (Albustix) [0-4]** | **age**  **[weeks]** |
| --- | --- | --- | --- | --- | --- |
| **rel. heart weight [g]** | **r= 0.346** (n=59)** | **r= 0.452** (n=43)** | **r= 0.672*** (n=43)** | **r= 0.592*****  **(n=57)** | r= -0.117  (n=59) |
| **wall thickness left ventricle [μm]** | **r= 0.323* (n=57)** | **r= 0.405** (n=43)** | **r= 0.345* (n=43)** | r= 0.045  (n=54) | r= 0.167  (n=57) |
| **wall thickness septum [μm]** | r= 0.192 (n=57) | r= 0.221 (n=43) | r= 0.264 (n=43) | **r= 0.373****  **(n=54)** | r= 0.015  (n=57) |
| **wall thickness intramyocardial arteries [μm]** | **r= 0.374** (n=57)** | **r= 0.368* (n=43)** | **r= 0.347* (n=43)** | **r= 0.296***  **(n=54)** | r= 0.058  (n=57) |
| **wall thickness aorta [μm]** | **r= 0.310* (n=54)** | r= 0.060 (n=41) | r= -0.054 (n=41) | r= -0.024  (n=51) | r= -0.174  (n=54) |

r: Spearman’s rank correlation; * p<0.05, ** p<0.01, ***p<0.001.

Supplemental Table 5. Pairwise correlation analyses of cardiovascular changes with cardiac expression of pro-inflammatory cytokines and factors in female NZB/W mice with lupus disease.

|  | **cardiac mRNA expression [∆∆CT-value]** | | | | | |  |
| --- | --- | --- | --- | --- | --- | --- | --- |
|  | **Ifi202b** | **IL-15** | **IL-6** | **IL-10** | **CRP** | **INFγ** | |
| **rel. heart weight [g]** | r= -0.214 (n=79) | r= -0.186 (n=87) | r= 0.209 (n=82) | r= -0.058  (n=87) | **r= 0.261***  **(n=81)** | r= -0.184 (n=78) | |
| **wall thickness left ventricle [μm]** | **r= 0.316** (n=74)** | r= 0.073 (n=83) | **r= 0.299** (n=75)** | r= 0.050  (n=83) | r= -0.169  (n=74) | r= -0.226 (n=72) | |
| **wall thickness septum [μm]** | **r= 0.335** (n=73)** | r= -0.038 (n=82) | **r= 0.301** (n=74)** | **r= 0.234***  **(n=82)** | **r= -0.282***  **(n=73)** | r= -0.129 (n=71) | |
| **wall thickness intramyocardial arteries [μm]** | r= 0.087 (n=87) | r= -0.093 (n=83) | **r= 0.364** (n=75)** | r= 0.166  (n=83) | r= 0.206  (n=74) | r= -0.108 (n=72) | |
| **wall thickness aorta [μm]** | **r= 0.295* (n=72)** | r= 0.052 (n=79) | r= -0.066 (n=73) | r= 0.158  (n=79) | r= -0.219  (n=73) | r= -0.183 (n=70) | |

r: Spearman’s rank correlation; * p<0.05, ** p<0.01, ***p<0.001.

**Supplemental Table 6: Comparison of cardiac and renal IFI202b and IL-6 expression in NZB/W mice using linear regression analysis.**

|  | Card. IFI202b expression [∆∆CT-value] | Renal  IFI202b expression [∆∆CT-value] | Card. IL-6 expression [∆∆CT-value] | Renal IL-6 expression [∆∆CT-value] |
| --- | --- | --- | --- | --- |
| Card. IFI202b expression [∆∆CT-value] | 1.000 | **0.942***** | 0.157 | 0.140 |
| Renal IFI202b expression [∆∆CT-value] | **0.942***** | 1.000 | 0.137 | 0.330 |
| Card. IL-6 expression [∆∆CT-value] | 0.157 | 0.137 | 1.000 | **0.592**** |
| Renal IL-6 expression [∆∆CT-value] | 0.140 | 0.330 | **0.592**** | 1.000 |

LV, left ventricular; LN, Lupus nephritis;Significant correlations analyzed by Pearson’s test were marked by asterisks. ** p<0.01; ***p<0.001. n=19


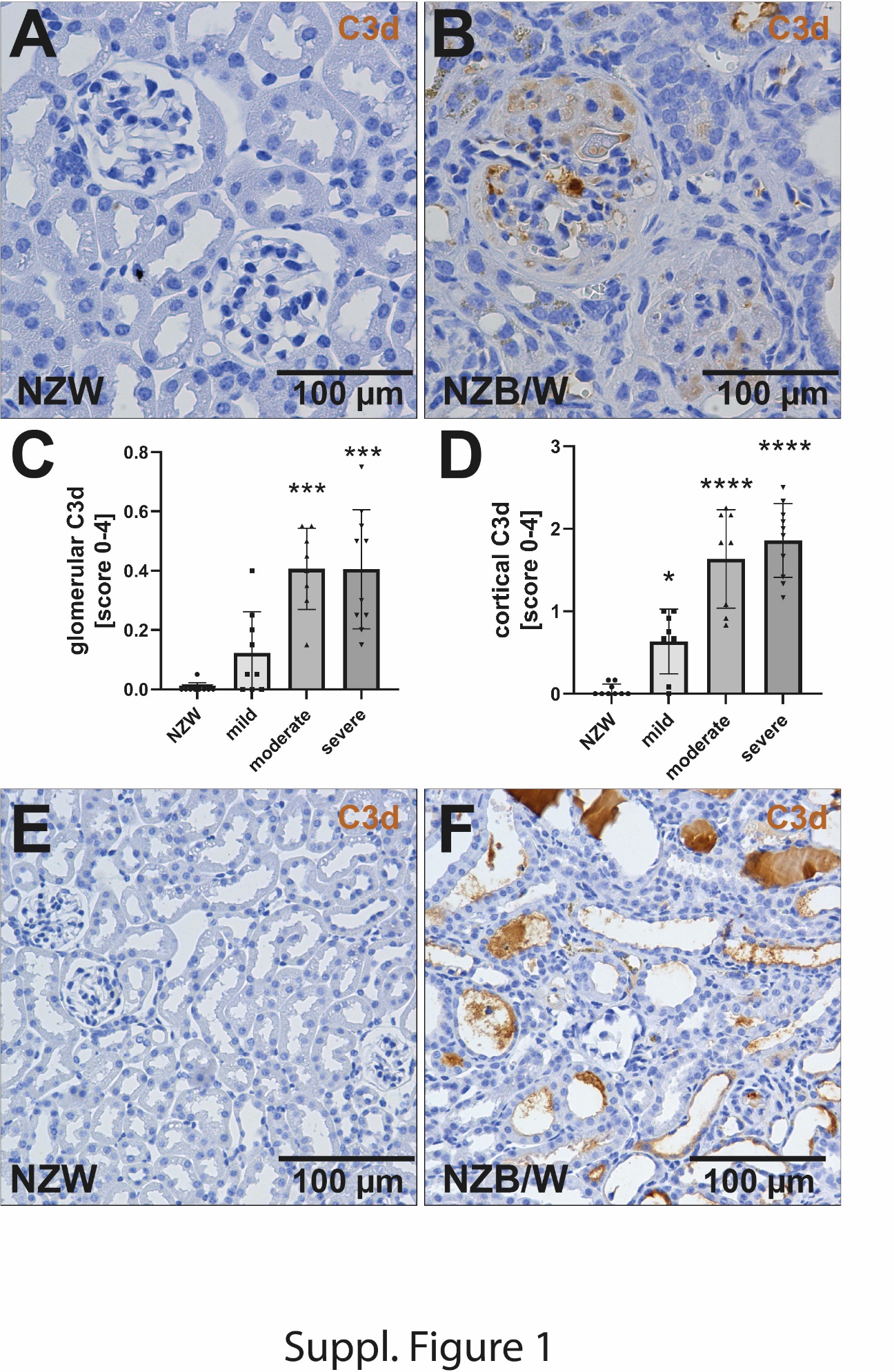


**Supplemental Figure 1: Renal deposition of complement C3d in the NZB/W lupus nephritis model.** Representative pictures of glomerular (A, B) and cortical area (E, F) in kidney sections of NZW (A, E) control and NZB/W mice (B, F) stained for C3d using immunohistochemistry are shown (brown staining). C3d deposition was analyzed using a semi-quantitative score in the glomerular (C) and tubulointerstitial compartment (D).
